# The complete mitochondrial genome of moonfish *Mene maculata* (Perciformes: Menidae)

**DOI:** 10.1080/23802359.2017.1407696

**Published:** 2017-11-27

**Authors:** Shengping Zhong, Yanfei Zhao, Xianfeng Wang, Zhifei Song, Qin Zhang

**Affiliations:** Key Laboratory of Marine Biotechnology, Guangxi Institute of Oceanology, Beihai, China

**Keywords:** Mitochondrial genome, *Mene maculata*, Menidae

## Abstract

The moonfish *M. maculata* is economic important species in the marine fishery in China. However, the genetic information of this species remains unavailable. In this study, we report the complete mitochondrial genome sequence of *M. maculata*. The mitogenome has 16,733 base pairs (53.8% A + T content) and made up of total of 37 genes (13 protein-coding, 22 transfer RNAs, and two ribosomal RNAs), and a putative noncoding control region. This study reports the first available complete mitogenome of Menidae and will provide useful genetic information for future fishery management and aquaculture development of Menidae.

Menidae are a morphologically distinctive group, which are easily recognized by their laterally compressed disc-like bodies and dorsally oriented mouth large (Friedman and Johnson 2005). The moonfish *M. maculata* is the single species belonging to the Menidae, which is distributed in the Indo-Pacific tropical waters from East Africa to southern Japan and northeastern Australia (Du et al. 2012). It has become an economic important species in Beibu Bay and its Fishing catch has been steadily increased in recent years. In spite of its commercial importance, adequate genetic information about this species and the genus is still missing. Here, we report the first complete mitochondrial genome sequence of *M. maculata* which will be an important genetic resource for stock management and genetic assessment.

A tissue sample of *M. maculata* was collected from GuangXi province, China (Beihai, 21.426285N, 109.260057E), and the whole body specimen (#GF0235) was deposited at Marine biological Herbarium, Guangxi Institute of Oceanology, Beihai, China. The total genomic DNA was extracted from the muscle of the specimens using an SQ Tissue DNA Kit (OMEGA, Guangzhou, China) following the manufacturer’s protocol. DNA libraries (350 bp insert) were constructed with the TruSeq NanoTM kit (Illumina, San Diego, CA) and were sequenced (2 × 150 bp paired-end) using HiSeq platform at Novogene Company (Beijing, China). Mitogenome assembly was performed by MITObim (Hahn et al. 2013). The cytochrome oxidase subunit 1 (COI) gene of *M. maculata* (GenBank accession number: KJ202178) was chosen as the initial reference sequence for MITObim assembly. Gene annotation was performed by MITOS (Bernt et al. 2013).

The complete mitogenome of *M. maculata* was found to be 16,733 bp in length (GenBank accession number: MG099701), and contains a typical set of 13 protein-coding, 22 tRNA, and two rRNA genes, and a putative control region. The overall base composition of the mitogenome was estimated to be A 29.2%, T 24.6%, C 30.5%, and G 15.8%, with a slightly high A + T content of 53.8%, which is similar, but slightly lower than *Hapalogenys nigripinnis* (56.8%) (Liang et al. 2012). The gene order in *M. maculata* is also highly similar to that found in *H. nigripinnis*, which indicated close relationship between *M. maculate* and *H. nigripinnis*. The result of phylogenetic tree of 15 species (including other 14 species from Suborder Percoidei in NCBI) also supported the close relationship between *M. maculate* and *H. nigripinnis* (Figure 1), as they shared the same branch node with the highest bootstrap value. All protein-coding genes were found to use the initiation codon ATG except for COX1 genes, where GTG served as the initiation codon. COX2, NAD4, and Cytb terminated with an incomplete stop codon T, which is thought to be completed with the addition of 3′ adenine residues to the mRNA (Ojala et al. 1981). The complete mitochondrial genome sequence of *M. maculate* was the first sequenced mitogenome within the family Menidae, which will contribute to further phylogenetic and comparative mitogenome studies of the family Menidae and related families.

**Figure 1.**
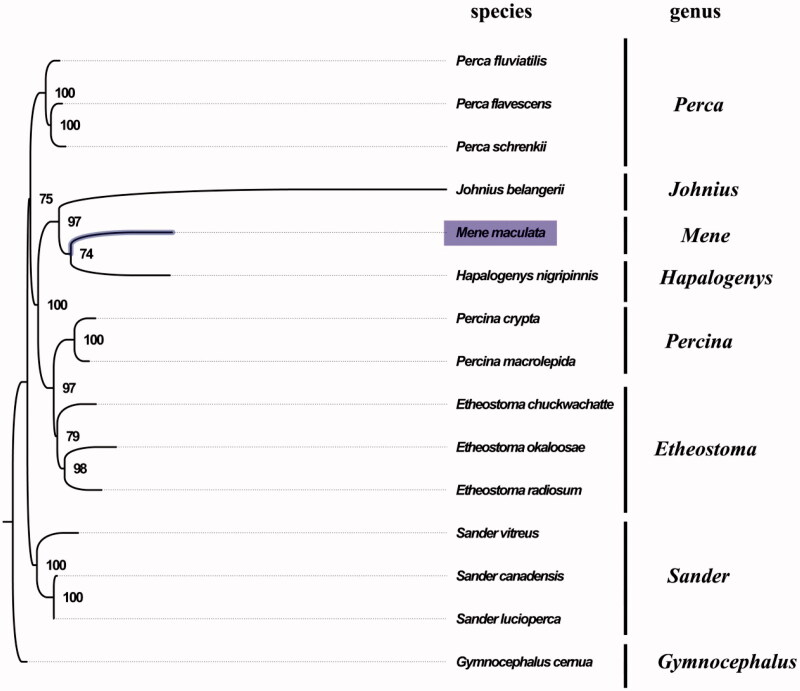
Phylogenetic tree of 15 species in Suborder Percoidei. The complete mitogenomes are downloaded from GenBank and the phylogenic tree is constructed by maximum-likelihood method with 100 bootstrap replicates. The bootstrap values were labeled at each branch nodes. The gene's accession number for tree construction is listed as follows: *Perca fluviatilis* (NC_026313.1), *Perca flavescens* (NC_019572.1), *Perca schrenkii* (NC_027745.1), *Johnius belangerii* (NC_022464.1), *Hapalogenys nigripinnis* (NC_014404.1), *Percina crypta* (NC_035945.1), *Percina macrolepida* (NC_008111.1), *Etheostoma chuckwachatte* (NC_035943.1), *Etheostoma okaloosae* (NC_035493.1), *Etheostoma radiosum* (NC_005254.2), *Sander vitreus* (NC_028285.1), *Sander canadensis* (NC_021444.1), *Sander lucioperca* (NC_026533.1), and *Gymnocephalus cernua* (NC_025785.1).
